# Infigratinib for the Treatment of Metastatic or Locally Advanced Cholangiocarcinoma With Known FGFR2 Gene Fusions or Rearrangements

**DOI:** 10.7759/cureus.46792

**Published:** 2023-10-10

**Authors:** Kathryn White, Ahmed I Anwar, Kevin Jin, Victoria Bollich, Rucha A Kelkar, Norris C Talbot, Rachel J Klapper, Shahab Ahmadzadeh, Omar Viswanath, Giustino Varrassi, Sahar Shekoohi, Alan D Kaye

**Affiliations:** 1 School of Medicine, Louisiana State University Health Sciences Center, Shreveport, USA; 2 Department of Psychology, Quinnipiac University, Hamden, USA; 3 School of Medicine, Medical University of South Carolina, Charleston, USA; 4 Radiology, Louisiana State University Health Sciences Center, Shreveport, USA; 5 Department of Anesthesiology, Louisiana State University Health Sciences Center, Shreveport, USA; 6 Pain Management, Valley Pain Consultants - Envision Physician Services, Phoenix, USA; 7 Pain Medicine, Paolo Procacci Foundation, Rome, ITA

**Keywords:** cca, cholangiocarcinoma, infigratinib, targeted therapy, fibroblast growth factor receptor (fgfr)

## Abstract

Cholangiocarcinoma (CCA) is an aggressive and diverse malignancy with a poor prognosis. Related to a typical indolent course of progression, most cases of CCA are metastatic or locally advanced at the time of presentation. For patients with nonresectable tumors or metastatic disease, the mainstay of treatment is comprehensive with combination chemotherapy. The first-line chemotherapeutic combination for the treatment of CCA are cisplatin and gemcitabine-based chemotherapies. However, many locally advanced and progressive CCA cases are refractory to first-line management. Within the past few years, the increase in the incidence of metastatic CCA and its poor prognosis has brought to light the need for novel therapeutic approaches to treatment. With advancements in next-generation genome sequencing, multiple molecular pathways have been identified in the pathogenesis of CCA and have shown great potential as alternative treatments in cases of CCA refractory to surgical resection. FGFR2 fusions or rearrangements have been identified in 10-16% of all intrahepatic CCA and are thought to serve as a pathway of resistance for a number of nonresectable and refractory cases of cholangiocarcinoma. A novel therapeutic agent that has been discussed is infigratinib, a selective, ATP-competitive inhibitor of fibroblast growth factor receptor 2 (FGFR2). In a phase 1 trial, infigratinib showed a safe profile and showed remarkable clinical efficacy in advanced CCA with FGFR2 fusions or rearrangements in phase II trials. As of May 2021, the Food and Drug Administration (FDA) approved infigratinib for CCA largely based on tumor response and duration of response. As of 2021, infigratinib, futibatinib, and pemigatinib, similar novel selective FGFR inhibitors, have been approved by the FDA for the treatment of locally advanced or metastatic CCA harboring *FGFR2* gene mutations. The present investigation reviews the development of infigratinib in particular and its clinical efficacy compared to other available treatment options for cholangiocarcinoma. While the side effect profile of infigratinib is minimal, particularly GI side effects, when compared with futibatinib and pemigatinib, the overall response rate (ORR) and median overall survival (mOS) for infigratinib (ORR=23.1%, mOS=3.8 months) was significantly lower than futibatinib (ORR=35.8%, mOS=21.1 months) and pemigatinib (ORR=35.5%, mOS=21.1 months). While there is ample promise for the use of infigratinib as molecular-directed therapy in the treatment of CCA harboring FGFR2 mutations, there is an appropriate concern for patient-acquired resistance. The heterogeneous nature of FGFR mutations and the emergence of different resistance mechanisms emphasize a need for more agents to inhibit FGFR rearrangements effectively.

## Introduction and background

Cholangiocarcinoma (CCA) develops from the malignant transformation of biliary epithelial cells in various areas of the biliary tract, including the intrahepatic, perihilar, and extrahepatic bile ducts [1]. CCA is divided into extrahepatic cholangiocarcinoma (eCCA), intrahepatic cholangiocarcinoma (iCCA), perihilar cholangiocarcinoma (pCCA), and distal cholangiocarcinoma (pCCA). Recent studies have shown that approximately 50% of cases of CCA are perihilar, 40% are distal, and 10% are intrahepatic [1]. Given its often silent course of progression, many cases of CCA are metastatic or locally advanced at the time of presentation [1].

While different subgroups of CCA (eCCA, iCCA, pCCA, and dCCA) differ in prognosis, etiology, biology, and epidemiology, surgical resection is the optimal management for the majority of localized cases of cholangiocarcinoma [2]. However, most cases of CCA (up to 75%) are identified as metastatic or locally advanced at the time of diagnosis, due to a lack of early clinical symptoms. Thus, nonresectable CCA is primarily treated with comprehensive therapeutic approaches, depending mainly on gemcitabine and cisplatin-based combination chemotherapies while also considering pain management strategies for advanced tumors [3]. Furthermore, maintenance of patient pain and proper analgesic support relies on proper analysis of the current development of CCA, its risk of growth in the future, its pain effects on the body, addiction factors, and the patient’s psychological distress and history of pain [4]. In the USA, the incidence of intrahepatic CCA increased between 2000 and 2015, with an annual percentage change of 5.06%, and an estimated 8000 new cases of CCA are diagnosed annually [5-6]. The increased incidence of advanced cholangiocarcinoma, poor prognoses, and subsequent sparse treatment options emphasize a need for novel therapeutic agents. As a growing public health concern, this creates a window of opportunity for the emergence of targeted therapy to become one of the most innovative approaches to therapy. As the role of molecular pathways in malignancy progression has become better understood, targeted therapy has been trialed to attack particular genes or proteins playing crucial roles in the carcinogenesis and progression of CCA. One molecular target of interest in advanced CCA is the fibroblast growth receptor 2 (*FGFR2*) gene. Studies show that advanced CCA with known *FGFR2 *gene fusions are not effectively treated with the available second or later-line chemotherapies and would benefit from molecular targeted therapy. Three orally bioavailable, potent, selective ATP-competitive inhibitors of FGFR, pemigatinib, infigratinib, and futibatinib, have received FDA approval as treatment options for advanced CCA [7]. These three therapies have shown anti-tumor activity and manageable safety against tumors with FGFR alterations in early clinical studies and are currently undergoing phase III clinical trials [8]. The present investigation reviews the anti-tumor activity of infigratinib in patients with locally advanced or metastatic CCA compared to currently available treatments including first-line therapy with gemcitabine and cisplatin as well as other selective FGFR2 inhibitors, pemigatinib, and futibatinib.

## Review

Methods

This was a narrative review. An extensive search of studies related to the use of FGFR2 inhibitor infigratinib for the treatment of nonresectable CCA within the PubMed, Google Scholar, Medline, and ScienceDirect databases was conducted. To identify relevant articles, we employed a comprehensive search strategy incorporating keywords such as “cholangiocarcinoma,” “fibroblast growth factor receptor (FGFR),” “targeted therapy,” and “infigratinib.” The selected studies encompassed various study types including randomized controlled trials, literature reviews, systematic reviews, and meta-analyses. Our preference for these study types was driven by their capacity to offer the most current and comprehensive insights available in the literature pertaining to the use of FGFR2 inhibitors in the treatment of nonresectable, advanced cholangiocarcinoma. Sources were accessed between April 2023 and August 2023.

Overview of cholangiocarcinoma (CCA)

CCA is a rare yet aggressive malignancy and is the second most common primary hepatic malignancy [9]. CCA comprises around 3% of gastrointestinal malignancies and accounts for approximately 10-15% of all hepatobiliary malignancies [10]. The incidence of CCA increases with age, and men are slightly more likely to be diagnosed than women [11]. The incidence rate of primary CCA ranges from 0.35 to 2 per 100,000 people every year in Western countries. However, this rate has become as much as 40 times greater in China [9]. Like other malignancies, CCA arises from precursor lesions in the normal biliary epithelium of intrahepatic or extrahepatic bile ducts. These lesions are acquired through oncogenic and tumor suppressor gene mutations in the normal epithelium, such as RAS, BRAF, p52, SMAD4, and more [12,13]. Approximately 90-95% of CCA are adenocarcinomas, while the remaining are squamous cell carcinomas [14]. They are subtyped into sclerosing, nodular, and papillary, depending on morphology. The presentation of CCA depends on the tumor location; however, presenting symptoms are often vague and non-localizable. Intrahepatic CCAs are usually asymptomatic, while extrahepatic CCAs may obstruct the biliary system leading to jaundice, pruritis, clay-colored stools, and dark-colored urine. Most patients present with generalized symptoms such as abdominal pain, weight loss, fever, fatigue, and night sweats. Diagnosis depends on the suspected location of the lesion. Given the often-nonspecific presentation, it is important to consider CCA in the differentials in patients with signs of biliary obstruction or primary sclerosing cholangitis.

At present, surgical resection, therefore, is the only effective form of treatment for localized cholangiocarcinoma. Intrahepatic (iCCA) is often treated with a hepatectomy, while extrahepatic (eCCA) may be treated with a hepatopancreatoduodenectomy. However, the surgical recurrence rate of primary resected CCA can be >50% despite receiving postoperative adjuvant chemotherapy [7]. About 75% of CCA patients are identified as metastatic or locally advanced disease at initial diagnosis and are not amendable to surgical resection due to a lack of early clinical symptoms. Cisplatin/gemcitabine (CisGem) is considered the first-line treatment for patients with advanced CCA [9]. However, it is believed that systemic chemotherapy’s overall effect on treating CCA remains unsatisfactory.

The universal first-line therapy for the treatment of CCA, according to the ABC 02 study, is cisplatin/gemcitabine (CisGem) combination chemotherapy. While there are available treatment options, many patients with CCA progressing after first-line therapy have limited treatment options [15]. According to the ABC 06 study, the standard second-line therapy following a failed trial of CisGem is fluorouracil plus oxaliplatin (FOLFOX). However, the objective response rate was not statistically significant between the FOLFOX and control groups [15]. Additionally, in the phase 3 SWOG 1815 trials, adding nab-paclitaxel, a taxane-based chemotherapy, to CisGem therapy did not significantly improve the OS of patients with newly diagnosed advanced CCA [16]. Thus, systemic chemotherapy provides only a modest benefit in most cases of advanced cholangiocarcinoma.

New advancements in genomic mapping have provided an enhanced understanding of the molecular pathways involved in the pathogenesis of different malignancies. In a previous study where next-generation sequencing (NGS) was used for mapping CCA, 182 cancer-associated genes and 37 introns were identified from 14 cancer-rearranged genes, which demonstrated that biliary tract tumors share the same chromatin remodeling (ARID1A) and genomic aberrations (CDKN2B) [17]. The tumor microenvironment (TME) of CCA contains tumor-associated fibroblasts and inhibitory immune components, leading to T-cell-mediated rejection, inhibition of anti-tumor immune response, and promotion of tumorigenesis, as well as possibly influencing the mechanism of chemotherapy [18].

*FGFR2* gene rearrangements and their role in advanced CCA

One genetic fusion of interest in investigating targeted therapy for advanced CCA is FGFR 2 rearrangements. FGFRs are receptor tyrosine kinases that regulate cell proliferation and differentiation, embryogenesis, angiogenesis, survival, organogenesis, migration, and wound repair. Gene fusions that result in constitutively active FGFR signaling have been indicated as a molecular driver in developing specific CCA subtypes [7]. FGFR2 fusions or rearrangements occur in 10-16% of patients with intrahepatic cholangiocarcinoma (iCCA) [7]. Second-line or later-line chemotherapy has limited efficacy in locally advanced or metastatic CCA with a known FGFR fusion or gene rearrangement. Preclinical studies have demonstrated the anti-tumor efficacy of FGFR inhibition selectively in cells harboring FGFR2 gene fusions [19,20]. Moreover, BGJ398, a pan-FGFR inhibitor, significantly reduced tumor burden in a genetic murine model of CCA and a patient-derived xenograft model of iCCA [21]. This data supports the notion that patients who carry FGFR2 gene fusions may benefit from FGFR-directed therapy in a precision-based chemotherapeutic approach. Early clinical studies have primarily focused on using non-selective FGFR2 inhibitors in patients with CCA and other solid malignancies with a known FGFR2 mutation. Non-selective FGFR inhibitors have short-term responses as well as disappointing clinical outcomes. Thus, selective FGFR inhibitors have been the clinical research and testing focus. Pemigatinib, an ATP-competitive selective FGFR1-4 inhibitor, has been shown to significantly extend survival in the second-line setting to over 20 months in patients who harbor FGFR2 fusions [22]. Futibatinib, an irreversible FGFR1-4 inhibitor, has also demonstrated efficacy among previously treated patients with intrahepatic cholangiocarcinoma (iCCA) with FGFR2 fusions/rearrangements in the FOENIX-CCA2 (NCT02052778) pivotal phase 2 trial [23].

Infigratinib, aka Truseltiq, approval for treating advanced cholangiocarcinoma

IInfigratinib is an ATP-competitive, selective FGFR tyrosine kinase inhibitor that was approved on May 28, 2021, to treat unresectable metastatic cholangiocarcinoma. FGFR plays a key role in promoting angiogenesis and the subsequent proliferation of cancerous cells [24].

Pharmacodynamics 

Cancerous cells activate FGFR via mutations or amplifications to proliferate. Infigratinib targets and suppresses FGFR1, FGFR2, and FGFR3, which have an IC50 of 1.1 nM, 1 nM, and 2 nM, respectively. Infigratinib has a mechanism of action that classifies it as a reversible ATP-competitive, selective FGFR1-3 inhibitor [25]. Blocking these receptors blocks the downstream pathways of RAS-MAPK and PI3K-AKT to decrease proliferation and induce apoptosis. Xenograft models of cholangiocarcinoma with infigratinib have shown that it activates FGFR2 and FGFR3 alterations [24].

Pharmacokinetic

Infigratinib is an oral drug with a rapid absorption rate and peak plasma concentration of 2 hours. It has a bioavailability estimated at around 75% and a plasma protein binding approximated at 99%. The mean steady-state maximum drug concentration (Cmax) from 0 to 24 hours is estimated at 282 ng/mL [24]. The mean Cmax was found to be increased after a high-fat and high-calorie meal by 70% and 100%, respectively [24]. The steady state is approximated to be 15 days with a median time to reach Cmax of 6 hours and mean apparent volume of distribution estimated to be 1600 L. Infigratinib is metabolized by CYP3A4, and its major metabolites are BHS697 and CQM157, which account for 16-33% and 9-12% respectively. Radiolabeled doses showed ~77% excretion via feces and 7.2% via urine [24].

Clinical Profile and Efficacies

A phase I basket trial of 132 patients with FGFR2 malignancies, including lung, breast, bladder, and colon, showed a maximum tolerated dose of 125 mg once daily in a continuous 28-day cycle. The primary adverse effect noted was hyperphosphatemia, followed by moderate increases in ALT/AST and corneal toxicity [25]. These side effects became less frequent after switching to a 3-week-on and 1-week-off cycle, as the median time to first dose interruption was found to be 22 days [26]. After switching, the safety profile was found to be tolerable, with the most prevalent side effect continuing to be hyperphosphatemia (74.2%), followed by constipation (40.2%) and poor appetite (40.2%) [25]. Hyperphosphatemia indicates that FGFR is inhibited, as it also plays a role in “FGF23-mediated phosphate homeostasis” [25].

In a phase II single-arm clinical trial (NCT02150967), 61 patients with CCA refractory to gemcitabine were given infigratinib. The median treatment duration was 4.7 months, and nine patients, all with FGFR2 fusions, showed a partial response, while 37 patients showed stable disease [25]. The ORR was 14.8%, and the median progression-free survival (PFS) was found to be 5.8 months. However, 50 patients did discontinue therapy, most secondary to progressive disease [25]. An updated study of *FGFR2 *gene fusions and rearrangements of 108 patients, with 83 (77%) patients specifically having an FGFR2 fusion, was conducted and showed the ORR to be 23.1% and PFS to be 7.3 months. Due to this increasing efficacy shown in the phase 2 trial, the FDA approved infigratinib in May 2021 [25]. However, patients do acquire resistance over time [27]. According to a case study by Krook et al., a patient enrolled in the phase II trial of infigratinib initially responded to treatment as evidenced by imaging and tumor marker decreases [28]. However, after 8 months of trial, disease progression was shown and found to have a sequence of FGFR2 kinase domain p.E565A and p.L17M single nucleotide variants (SNV). These SNVs are hypothesized to upregulate the PI3K/AKT/mTOR pathways and re-sensitize cells to FGFR inhibition [28]. It has been hypothesized that combining infigratinib with other FGFR and mTOR inhibitors in combination therapy may offer better success [28].

Efficacy of alternative treatments for advanced cholangiocarcinoma and special considerations

Current guidelines state that gemcitabine plus cisplatin is the first-line regimen for patients with locally advanced CCA [29]. Gemcitabine is a cytidine analog that disrupts DNA synthesis in rapidly dividing cells, such as cancer cells. After gemcitabine is taken up by the cancer cell, it is phosphorylated initially by deoxycytidine kinase, then further phosphorylated by other enzymes, forming its active diphosphate (dFdCDP) and triphosphate (fDCTP) metabolites. These metabolites are then integrated into DNA and RNA, halting synthesis. Most of gemcitabine is metabolized by the liver, and some in the blood, by removal of an amine group to form 2′,2′‐difluoro‐2′‐deoxyuridine (dFdU) [30]. Furthermore, 90% of an IV dose of gemcitabine can be found excreted in the urine, most of it being made up of dFdU [30]. Gemcitabine reaches a plasma steady state after 15-30 mins with a standard infusion of over 30 minutes and has a half-life between 2 and 24 hours [30]. Compared to gemcitabine alone, gemcitabine, combined with cisplatin, was shown to have a significant survival advantage in treating advanced biliary tract cancer [31]. As shown in the ABC-02 trial, patients who received gemcitabine plus cisplatin lived an average of 3.6 months longer than with gemcitabine alone, with a median OS of 11.7 months [31]. In an earlier study, the ABC-01 trial, patients with combination therapy showed increased grade 3 or 4 fatigue, but this adverse reaction was not seen in the ABC-02 trial [32]. Additionally, there was a significant decrease in liver function in the gemcitabine-only group compared to the combination group (27.1% vs 16.7%) [31].

Pemigatinib was FDA-approved in 2020 for treating previously treated unrespectable advanced CCA with FGFR2 alterations. Pemigatinib is a selective inhibitor of FGFR1-3 with weak activity against FGFR4 that can be taken orally [33]. FGFR2 alternations have been almost exclusively isolated to intrahepatic cholangiocarcinoma, and the prevalence of this alteration ranges from 10-15% [22]. Since this medication spares FGFR4, there is a decrease in hepatotoxicity [22]. The FIGHT-202 trial enrolled 146 patients, 107 with FGFR2 alterations, and showed that 38 (35.5%) patients achieved an objective response to the treatment [34]. The other two cohorts, patients with other FGFR alterations and patients without FGFR alterations, had an objective response [34]. The median OS was 21.1 months, more significant than the 11.7% in the gemcitabine/cytidine combination [22]. The most common adverse effects were hyperphosphatemia (60%), hypophosphatemia (12%), and arthralgia (6%) [22].

Futibatinib is an oral FGFR1-4 selective inhibitor that was FDA-approved in 2022 for treating locally advanced or metastatic CCA [33]. Futibatinib irreversibly binds to FGFR using covalent bonds, making it less susceptible to mutations that can cause resistance. The FOENIX-CCA2 showed that 43 out of the 103 patients who received futibatinib had an achievable response [35]. The median follow-up was 17.1 months, with a PFS of 9.0 months. The OS was 21.7 months, comparable with pemigatinib. The most common adverse effects included hyperphosphatemia (30%), increased aspartate aminotransferase level (7%), and stomatitis (6%) (Table 1) [35].

**Table 1:** Efficacy of various treatments for cholangiocarcinoma [24-26]

	Mechanism of action	Overall response rate (ORR)	Median overall survival (mOS)	Median progression-free survival (PFS)	Adverse effects
Infigratinib	FGFR tyrosine kinase inhibitor	23.1%	3.8 months	5.8 months	Hyperphosphatemia (74.2%), constipation (40.2%), poor appetite (40.2%)
Gemcitabine + Cisplatin	Cytidine analog	19.5%	11.7 months	5.8 months	Neutropenia (56.1%), thrombocytopenia (39.0%), leukopenia (29.3%)
Pemigatinib	ATP-competitive selective inhibitor of FGFR1-3	35.8%	21.1 months	6.9 months	Hyperphosphatemia (60%), alopecia (46%), dysgeusia (38%)
Futibatinib	FGFR1-4 selective, irreversible inhibitor	35.5%	21.1 months	6.9 months	Hyperphosphatemia (30%), increased aspartate aminotransferase level (7%), stomatitis (6%)

Based on the literature review, infigratinib showed fewer incidences of AEs compared to other agents (including pemigatinib or futibatinib) in diarrhea (15% vs 21-37%), nausea (15% vs 25%-45%), or decreased appetite (12% vs 17-24%) [25].

Limitations to this study, which are inherent to many narrative reviews, include a high susceptibility to reviewer bias, objectivity of data collection, completeness of literature review, and interpretation of findings. Additionally, there was no standardized protocol to guide this review.

## Conclusions

In addition to pemigatinib and futibatinib, infigratinib is one of the most clinically advanced and investigated FGFR inhibitors in CCA management as they have promising clinical activity in previously treated patients with locally advanced or metastatic CCA with known *FGFR2 *gene fusions or rearrangements. In addition, the side effect profile of infigratinib was manageable with fewer GI side effects when compared to other FGFR inhibitors. While there is ample promise for the use of infigratinib as molecular-directed therapy in the treatment of CCA harboring FGFR2 mutations, there is an appropriate concern for patient-acquired resistance. The heterogeneous nature of FGFR mutations and the emergence of different resistance mechanisms emphasize a need for more agents to inhibit FGFR rearrangements effectively. While treatment resistance is inevitable in advanced malignancy, additional combination therapy may improve clinical outcomes and median OS. Thus, patient-focused synergy is needed to solve the current therapeutic challenges.
